# Associations between omega-3 fatty acids and insulin resistance and body composition in women with polycystic ovary syndrome

**DOI:** 10.3389/fnut.2022.1016943

**Published:** 2022-10-05

**Authors:** Ling Lu, Xiaoqin Li, Lin Lv, Yao Xu, Baohua Wu, Chaolin Huang

**Affiliations:** ^1^Department of Gynecology, First Affiliated Hospital of Chengdu Medical College, Chengdu, China; ^2^Department of Oncology, Guangdong Second Provincial General Hospital, Guangzhou, China

**Keywords:** polycystic ovary syndrome, omega-3 polyunsaturated fatty acids, case-control studies, insulin resistance, body composition

## Abstract

**Background:**

Polycystic ovary syndrome (PCOS) is strongly associated with abdominal obesity and insulin resistance and effective approaches to nutrition (e.g., omega-3 fatty acids intake) might improve the cardiometabolic risk profile. This study aimed to examine the associations of dietary and serum omega-3 fatty acids with insulin resistance (IR) and body composition among PCOS patients.

**Methods:**

A total of 185 patients with PCOS were included in our analysis. Dietary information was collected through face-to-face interviews using a 102-item food frequency questionnaire (FFQ). Serum omega-3 fatty acid levels were measured with the gas chromatography method. Body composition was measured by both dual-energy X-ray absorptiometry (DXA) and bioelectrical impedance (BIA) methods. The multivariable linear regression model was applied to analyze the associations of dietary and serum omega-3 fatty acids with the levels of Homeostasis Model Assessment for Insulin Resistance (HOMA-IR) and body composition parameters among PCOS patients.

**Results:**

Our results indicated that the dietary long-chain omega-3 polyunsaturated fatty acids (PUFA) intakes were negatively associated with HOMA-IR (β = –0.089, *P* = 0.040), fat mass (β = –0.022, *P* = 0.047), and body fat percentage (β = –0.026, *P* = 0.032). For serum biomarkers, higher total omega-3 PUFAs levels (β = –0.158, *P* = 0.021) and long-chain omega-3 PUFAs levels (β = –0.187, *P* < 0.001), particularly eicosapentaenoic acid (EPA) (β = –164, *P* = 0.011) and docosahexaenoic acid (DHA) (β = –0.158, *P* = 0.001) were also associated with decreased HOMA-IR. In addition, generally, dietary and serum long-chain omega-3 PUFA levels, DPA, and DHA levels were both positively associated with muscle mass measured by DXA; whereas serum total, long-chain and individual omega-3 PUFA levels (e.g., DPA, EPA, and DHA) were all negatively associated with fat mass and body fat percentage. These findings were further confirmed by the findings for body composition measured by the BIA method.

**Conclusion:**

Higher levels of dietary and serum omega-3 PUFAs, particularly long-chain omega PUFAs (DPA and DHA), might have beneficial effects on metabolic parameters and body composition among PCOS patients.

## Introduction

Characterized by a series of manifestations of hyperandrogenemia, persistent anovulation, and ovarian polycystic changes (1), polycystic ovary syndrome (PCOS) is one of the most common endocrine-metabolic disorders that affects 5 to 10% of women of reproductive age (2). PCOS entails diseases such as type 2 diabetes, hypertension, cardiovascular disease, and endometrial cancer (3, 4), and insulin resistance (IR) and hyperandrogenemia might be closely associated with the pathogenesis of PCOS (5). Excessive insulin level is not only a potential cause of hyperandrogenemia but also one of the high-risk factors leading to metabolic syndromes among those with PCOS (6). PCOS can also be accompanied by obesity because central obesity could exaggerate IR and lead to hyperinsulinemia (7). Seventy percentage of overweight PCOS patients were accompanied by IR, with the majority of overweight PCOS patients also being accompanied by hyperinsulinemia (8), suggesting potential relationships between body fat with PCOS.

The etiology of PCOS is not yet clear, but genetic, environmental and lifestyle factors may contribute to its development (9, 10). Lifestyle intervention can effectively alleviate the pathological manifestations of abnormal reproduction and metabolism in patients with PCOS, and dietary intervention may also exert important effects on the prevention and control of PCOS and its complications (11, 12). Ensuring a certain intake level of dietary polyunsaturated fatty acids (PUFAs) might help improve the clinical manifestations of dyslipidemia, impaired vascular endothelial function, and IR in PCOS patients (11). The omega-3 fatty acids and omega-6 fatty acids are mainly dietary sources of the essential PUFAs (6), but the dietary sources of omega-3 fatty acids are relatively less abundant than omega-6 fatty acids, only found in plant foods (e.g., green leafy vegetables, vegetable oils, and seeds) and deep-sea fish (6). Particularly, the role of long-chain omega-3 PUFAs (eicosapentaenoic acid, EPA; docosahexaenoic acid, DHA) in the primary prevention of cardiovascular diseases (13), metabolic syndrome (adiposity, dyslipidemia, insulin resistance, and diabetes, etc.) have been reported before (14).

A growing number of evidence have shown that omega-3 PUFAs have been used to improve lipid and insulin profiles in inflammation and obesity (15–17). Omega-3 fatty acids are involved in the regulatory process of arachidonic acid derivatives in the human body, thereby down-regulating the expression levels of transcription factors related to inflammation in cells (18). An omega-3 fatty acids treatment could effectively enhance systemic insulin sensitivity in mice by up-regulating the function of G protein-coupled receptor 120 (GPR120) (19). A meta-analysis indicated that omega-3 fatty acids supplements have also been found to exert an anti-obesity effect on humans by reducing body fat (weight, waist circumference) (20). The chemical structures and effects of omega-3 PUFAs might vary between dietary and serum sources, but rare studies investigated the effects of dietary and serum omega-3 PUFA levels on IR and body composition in PCOS patients, respectively.

Hence, the purpose of this study is to evaluate whether dietary and serum of different types of omega-3 PUFAs are associated with IR and body composition among patients with PCOS.

## Materials and methods

### Assessment of dietary omega-3 fatty acid intakes

The assessment of omega-3 fatty acids in diet was conducted using a quantitative food frequency questionnaire (FFQ), which was adapted from the 2002 China National Nutrition and Health Survey capturing the usual intake of nutrients and major foods from Chinese (21). Based on the dietary habits of the Sichuan population, a total of 102 items were included in our FFQ. The main food items included cereals and their products, beans, vegetables, fruits, fungi and algae, nuts, livestock meat, poultry, dairy products, eggs, fish and seafood, and condiments. The Spearman correlation coefficients between the FFQ and 3-d dietary records for different fatty acid intakes were calculated among 24 local participants, and the results ranged from moderate to good (*r* = 0.28–0.59). The participants were interviewed through a face-to-face interview by trained researchers to recall their food consumption over the past year before PCOS diagnosis. The participants were asked to report the frequency (none, daily, weekly, monthly, and yearly) and portion size of consumption for each food item. Dietary intakes of individual omega-3 fatty acids included ALA (18:3 omega-3), EPA (20:5 omega-3), docosapentaenoic acid (DPA, 22:5 omega-3), and DHA (22:6 omega-3) were measured in this study.

### Measurement of serum omega-3 fatty acids

According to a previously published approach (22), blood fatty acid concentrations were determined using gas chromatography. Lipid extraction and subsequent gas chromatography analysis were used to assess the amounts of serum individual fatty acid levels. The Bligh et al. (23) method was used to extract and purify the total lipid from serum. An Agilent 6890A gas chromatograph (Agilent, Palo Alto, USA) and a capillary column (SP2380, 0.25 mm × 30 m, 0.25 μm film, Supelco, Bellefonte, PA) were used to separate fatty acid methyl esters. 1, 2-dinonadecanoyl-sn -glycerol-3-phosphocholine (C19:0) was regarded as an internal standard. A total of 28 fatty acids were quantified and the percentage composition of fatty acid methyl esters was calculated and normalized to 100%. The intra-assay coefficients of variation (CV) for the total omega-3 PUFA and individual omega-3 PUFA were 7.8 and 5.2~9.7%, respectively. All assays were performed at the laboratory of the First Affiliated Hospital of Chengdu Medical College.

### Measurement of body composition

In this study, we have body composition measured by both dual-energy X-ray absorptiometry and a body composition analyzer.

Dual-energy X-ray absorptiometry (DXA; GE-lunar Prodigy, Wisconsin, USA) scans were used to measure body composition. Total lean mass, total fat mass (FM), and body fat percentage (FM%) were automatically analyzed using the software V.enCORE10.50.086. According to the manufacturer's instructions, daily quality assurance scans were conducted by scanning the spine phantom. During the test, the subjects should keep quiet and prohibit strenuous or confrontational movements. The coefficient of variation was <2% for LM, FM, and FM% by duplicate scans with 18 volunteer subjects.

A body composition analyzer (InBodyS10) was based on the principle of BIA. The analyzer is an eight-electrode, multi-frequency (1, 50, 100, 250, and 500 kHz), 800 μA alternating current, which measures the human body in sections and measures the resistance and reactance at the same time to obtain the proportions of muscle and fat tissues of the limbs and trunk. Inter-individual coefficients of variations were 2.8% for muscle mass and 2.1% for fat mass among 35 randomly selected duplicates.

### Participant recruitment

A cross-sectional study was conducted from July 15, 2016, to July 28, 2019. Potentially eligible patients who suffered PCOS were consecutively recruited from the Department of Gynecology, the First Affiliated Hospital of Chengdu Medical College in China. The detailed information on the study design has been described in a previous publication (24).

Based on the diagnostic criteria of the Rotterdam conference in 2003 (25), those would be diagnosed with PCOS if they meet at least two of the following items: (i) oligo menorrhea (interval between two menstrual periods more than 35 days) and/or chronic anovulation (no vaginal bleeding for at least 6 months); (ii) clinical signs (hirsutism by elevated Ferriman-Gallwey score) and/or biochemical (elevated testosterone or free androgen index) hyperandrogenism; (iii) and/or polycystic ovaries on an ultrasound exam (≥12 follicles measuring 2–9 mm in diameter, or ovarian volume >10 mL in at least one ovary). We excluded participants who (i) were pregnant; (ii) had other genetic or endocrine disorders; (iii) had significant changes in dietary habits in the past 1 year; (iv) had mental or other diseases that might affect the accurate answer of the questionnaire survey; (v) reported a daily energy intake <600 or >3,500 kcal/d (26).

The demographic characteristics and biochemical parameters of PCOS patients were obtained from medical records. We collected the demographic characteristics (age, body mass index (BMI), waist-hip ratio (WHR), age at menarche, education level), living habits (smoking, drinking, fish oil supplementation, and physical activity frequency), the biochemical parameters (systolic blood pressure (SBP), diastolic blood pressure (DPB), fasting glucose, fasting insulin, total testosterone, follicle-stimulating hormone (FSH), luteinizing hormone (LH) levels and PCOS phenotypes). The Homeostasis Model Assessment for Insulin Resistance (HOMA-IR) index was calculated using the formula: HOMA-IR= fasting glucose (mg/dL) × fasting insulin [μUI/mL (mg/dL)]/405 (27).

A total of 351 eligible patients were identified and 325 (92.6% response rate) patients were successfully interviewed for dietary habits and biological sample collection. All subjects signed informed written consent forms for inclusion before they participated in the study. The protocol was approved by the ethical committee of the First Affiliated Hospital of Chengdu Medical College (2016CYFYHEC025).

### Statistical analysis

Data were expressed as means and standard deviations (SDs) for normal disquieted continuous variables, or medians and interquartile ranges (IQRs) for skewed variables. To assess the associations of the omega-3 PUFAs with HOMI-IR and body composition (LM, FM, and FM%), the multivariable linear regression test was performed and the standardized beta-coefficients and corresponding standard errors (SEs) were computed. All statistical analyses were performed using SPSS22.0. All statistical tests were two-tailed, and statistical significance was considered at *P*-values < 0.05.

## Results

Table 1 summarizes the demographic characteristic and biochemical parameters of the study patients. After completing the FFQ, the serum omega-3 fatty acids assessment, and DIX measurements, the sample only included 185 PCOS patients with a mean age of 29.3± 6.45 years. Mean values of BMI, WHR, and age at menarche were 21.5 ± 3.36 kg/m^2^, 0.88 ± 0.72, and 13.4 ± 1.21 years, respectively. For biochemical parameters, the mean levels of SBP, DBP, fasting glucose, fasting insulin, and HOMA-IR were 116.8 ± 10.9 mmHg, 79.7 ± 7.6 mmHg, 5.21 ± 0.79 mmol/L, 12.2 ± 5.14 mIU/ml, and 1.44 ± 0.72 respectively. The majority of participants were non-smokers (95.7%) and non-drinkers (95.1%). The percentage of fish oil supplement users was only 11.4%. Most patients reported that exercising <1 time per week (56.2%). For PCOS-related parameters, the mediums and IQRs of FSH and LH were 6.77 (3.16, 8.75) U/L and 10.41 (6.32, 17.05) U/L, respectively. For PCOS phenotypes, 33.0, 29.2, 22.7 and 15.1% of PCOS patients suffered from polycystic ovarian morphology (PCOM) + hyperandrogenism (HA) + ovulatory dysfunction (OD), PCOM + HA, PCOM + OD and HA + OD, respectively. For body composition measured by DXA, the mean levels were 30.23 ± 3.12, 18.62 ± 3.66, and 33.01 ± 3.56 for muscle mass, fat mass, and body fat percentage, respectively.

**Table 1:** Demographic characteristics and biochemical parameters among PCOS cases.

	**Cases (*n* = 185)**
Age, years	29.3 ± 6.45
BMI, kg/m^2^	21.5 ± 3.36
WHR	0.88 ± 0.72
Age at menarche, year	13.4 ± 1.21
Education, *n* (%)	
Middle school or below	50 (27.0)
High school	72 (38.9)
College or above	63 (34.1)
Current smokers, *n* (%)	
Yes	8 (4.3)
No	177 (95.7)
Current alcohol drinkers, *n* (%)	
Yes	9 (4.9)
No	176 (95.1)
Users of fish oil supplements, *n* (%)	
Yes	21 (11.4)
No	164 (88.6)
Physical activity [*n* (%)]	
<1 time/week	104 (56.2)
1~3 times/week	57 (30.8)
>3 times/week	24 (13.0)
SBP, mmHg	116.8 ± 10.9
DBP, mmHg	79.7 ± 7.6
Fasting glucose, mmol/L	5.21 ± 0.79
Fasting insulin (mIU/mL)	12.2 ± 5.14
HOMA-IR	1.44 ± 0.72
Total energy intake, kcal/day	1,724.1 ± 609.7
Total testosterone, nmol/L^a^	1.51 (0.50, 2.98)
FSH, U/L ^a^	6.77 (3.16, 8.75)
LH, U/L ^a^	10.41 (6.32, 17.05)
Insulin resistance, *n* (%)	
Yes	42 (22.50%)
No	143 (77.50%)
Obesity, *n* (%)	
Yes	85 (46.20%)
No	100 (53.80%)
Usage of drugs, *n* (%)^b^	
Metformin	32 (17.30%)
Spironolactone	10 (5.20%)
Rosiglitazone	1 (0.42%)
Statins	36 (19.50%)
Hormones	39 (21.30%)
Phenotypes, *n* (%)	
PCO+HA+OD	61 (33.0)
PCO+HA	54 (29.2)
PCO +OD	42 (22.7)
HA+OD	28 (15.1)
Body composition by DXA	
MM	30.23 ± 3.12
FM	18.62 ± 3.66
Body fat percentage (FM%)	33.01 ± 3.56
Body composition by BIA	
MM	31.76 ± 6.12
FM	18.21 ± 6.91
Body fat percentage (FM%)	32.45 ± 6.21

PCOS, polycystic ovary syndrome; BMI, body mass index; WHR, Waist Hip Ratio; SBP, systolic blood pressure; DBP, diastolic blood pressure; HOMA-IR, Homeostasis Model Assessment for Insulin Resistance; FSH, follicle-stimulating hormone; LH, luteinizing hormone; PCO, polycystic ovarian morphology; HA, hyperandrogenism; OD, ovulatory dysfunction; MM, muscle mass; FM, fat mass.

^a^Data was expressed as median and interquartile ranges (IQRs).

^b^Numbers (percentages) of participants who used specific drugs.

As shown in Table 2, for dietary omega-3 PUFAs, the average daily dietary intake of total omega-3 PUFAs was 1.05 ± 0.42 g/d, and long-chain omega-3 PUFAs was 44.3 ± 17.1 g/d. Among them, the daily intake of EPA (19.8 ± 9.3 mg/d) was the highest, whereas the level of ALA was the lowest (1.02 ± 0.39). With regard to serum phospholipid omega-3 PUFAs, the total serum- and the long-chain omega-3 PUFA levels were 4.84 ± 1.96 and 4.81 ± 1.93, respectively. Among the categories of omega-3 PUFAs, the level of DHA (3.45 ± 1.63) was the highest, while its serum level in ALA was the lowest (0.07 ± 0.04).

**Table 2:** Dietary and serum phospholipid omega-3 polyunsaturated fatty acids among PCOS cases (*n* = 185).

	**Mean ±SD**	**Median (25th, 75th)**
**Dietary omega-3 PUFA intakes**		
Total omega-3 PUFAs (g/day)	1.05 ± 0.42	1.04 (0.53, 1.42)
Long-chain omega-3 PUFAs (mg/day) ^a^	44.3 ± 17.1	37.1 (14.6, 65.7)
ALA (g/day)	1.02 ± 0.39	1.00 (0.51, 1.30)
DPA (mg/day)	6.39 ± 3.51	4.30 (1.57, 9.23)
EPA (mg/day)	19.8 ± 9.3	17.5 (5.9, 30.7)
DHA (mg/day)	18.1 ± 10.7	14.2 (6.0, 28.1)
**Serum phospholipid omega-3 PUFAs, %**		
Total omega-3 PUFAs	4.84 ± 1.96	4.63 (2.15, 6.71)
Long-chain omega-3 PUFAs ^a^	4.81 ± 1.93	4.65 (2.12, 6.67)
18:3 omega-3	0.07 ± 0.04	0.06 (0.03, 0.08)
22:5 omega-3 (DPA)	1.01 ± 0.49	0.93 (0.41, 1.62)
20:5 omega-3 (EPA)	0.34 ± 0.15	0.31 (0.10, 0.55)
22:6 omega-3 (DHA)	3.45 ± 1.63	3.34 (1.35, 5.24)

PCOS, polycystic ovary syndrome; SD, standard deviation; PUFA, polyunsaturated fatty acids; ALA, alpha-linolenic acid (18:3 omega-3); DPA, docosapentaenoic acid (22:5 omega-3); EPA, eicosapentaenoic acid (20:5 omega-3); DHA, docosahexaenoic acid (22:6 omega-3).

^a^Long-chain omega-3 PUFAs=DPA+EPA+DHA.

By using the multivariable linear regression with covariates entered, Table 3 shows the associations between omega-3 PUFAs and HOMA-IR level in PCOS patients. As for dietary intake of omega-3 PUFAs, the long-chain omega-3 PUFAs were significantly negatively related to HOMA-IR (ß = –0.089, *P* = 0.040), whereas total omega-3 PUFAs, as well as each category of long-chain omega-3 PUFAs, was not related with HOMA-IR (all *P*-values > 0.05). In terms of the serum omega-3 PUFAs, total levels of omega-3 PUFAs (ß=-0.158, *P* = 0.021), long-chain omega-3 PUFAs (ß = –0.187, *P* < 0.001), DHA (ß = –0.158, *P* = 0.001) and EPA (ß = –0.164, *P* = 0.011) were all negatively associated with the HOMA-IR, while blood levels of ALA (*P* = 0.565) and DPA (*P* = 0.082) were not.

**Table 3:** The multivariable analysis between Omega-3 fatty acids and the level of HOMA-IR among PCOS patients.

	**Standardized coefficients β**	**SE**	***P*-value^a^**
**Dietary omega-3 PUFA intakes**			
Total omega-3 PUFAs (g/day)	−0.051	0.036	0.158
Long-chain omega-3 PUFAs (mg/day)^b^	−0.089	0.043	**0.040**
ALA (g/day)	−0.004	0.013	0.759
DPA (mg/day)	−0.035	0.056	0.533
EPA (mg/day)	−0.054	0.032	0.093
DHA (mg/day)	−0.064	0.034	0.061
**Serum phospholipid omega-3 PUFAs, %**			
Total omega-3 PUFAs	−0.158	0.068	**0.021**
Long-chain omega-3 PUFAs ^b^	−0.187	0.051	**<0.001**
18:3 omega-3	−0.045	0.078	0.565
22:5 omega-3 (DPA)	−0.091	0.052	0.082
20:5 omega-3 (EPA)	−0.164	0.064	**0.011**
22:6 omega-3 (DHA)	−0.158	0.047	**0.001**

PCOS, polycystic ovary syndrome; HOMA-IR, Homeostasis Model Assessment for Insulin Resistance; β, standardized linear regression coefficients; SE, standard error; PUFA, polyunsaturated fatty acids; ALA, alpha-linolenic acid (18:3 omega-3); DPA, docosapentaenoic acid (22:5 omega-3); EPA, eicosapentaenoic acid (20:5 omega-3); DHA, docosahexaenoic acid (22:6 omega-3).

^a^Statistically significant P-value < 0.05 in bold.

^b^Long-chain omega-3 PUFAs=DPA+EPA+DHA.

The associations between omega-3 PUFAs and body composition measured by DXA were presented in Table 4. For dietary intakes of omega-3 PUFAs, the total level of long-chain omega-3 PUFAs and DHA was negatively associated with fat mass and body fat percentage but positively associated with muscle mass (all *p* < 0.05). For the serum levels of omega-3 PUFAs, both total long-chain omega-3 PUFAs (ß = 0.024, *P* = 0.017), DPA (ß = 0.019, *P* = 0.036), and DHA (ß = 0.022, *P* = 0.029) were positively correlated with muscle mass. On the contrary, the levels of total omega-3 PUFAs (ß = –0.025, *P* = 0.037), long-chain omega-3 PUFAs (ß = –0.043, *P* < 0.001), DPA (ß = –0.025, *P* = 0.024), EPA (ß = –0.031, *P* = 0.005) and DHA (ß = –0.042, *P* < 0.001) were all significantly negatively correlated with fat mass, with the same associations were found between the serum levels of total omega-3 PUFAs (ß = –0.041), long-chain omega-3 PUFAs (ß = –0.061), DPA (ß = –0.031), EPA (ß = –0.045) and DHA (ß = –0.051) and body fat percentage (all *P*-values < 0.05). The findings for composition measured by DXA were generally confirmed by those measured by BIA (Table 1).

**Table 4:** Associations between Omega-3 fatty acids and body composition measured by DXA method among PCOS patients.

	**Muscle mass**			**Fat mass**			**Body fat percentage**		
	**Standardized coefficients β**	**SE**	***P* value^a^**	**Standardized coefficients β**	**SE**	***P* value^a^**	**Standardized coefficients β**	**SE**	***P* value^a^**
**Dietary omega-3 PUFA intakes**									
Total omega-3 PUFAs (g/day)	0.007	0.005	0.163	−0.012	0.010	0.232	−0.015	0.010	0.135
Long-chain omega-3 PUFAs (mg/day)^b^	0.019	0.006	**0.002**	−0.021	0.010	**0.037**	−0.028	0.012	**0.021**
ALA (g/day)	0.001	0.009	0.912	−0.003	0.006	0.618	−0.004	0.008	0.618
DPA (mg/day)	0.011	0.005	**0.029**	−0.017	0.010	0.091	−0.010	0.012	0.406
EPA (mg/day)	0.010	0.008	0.213	−0.013	0.011	0.239	−0.009	0.012	0.454
DHA (mg/day)	0.021	0.008	**0.009**	−0.020	0.008	**0.013**	−0.023	0.010	**0.023**
**Serum phospholipid omega-3 PUFAs, %**									
Total omega-3 PUFAs	0.010	0.009	0.268	−0.023	0.012	0.057	−0.041	0.013	**0.002**
Long-chain omega-3 PUFAs^b^	0.024	0.010	**0.017**	−0.043	0.011	**<0.001**	−0.061	0.012	**<0.001**
18:3 omega-3	0.003	0.007	0.669	−0.017	0.013	0.193	−0.016	0.015	0.288
22:5 omega-3 (DPA)	0.019	0.009	**0.036**	−0.025	0.011	**0.024**	−0.031	0.012	**0.011**
20:5 omega-3 (EPA)	0.021	0.011	0.058	−0.031	0.011	**0.005**	−0.045	0.013	**0.001**
22:5 omega-3 (DHA)	0.022	0.010	**0.029**	−0.042	0.010	**<0.001**	−0.051	0.011	**<0.001**

PCOS, polycystic ovary syndrome; SE, standard error; PUFA, polyunsaturated fatty acids; ALA, alpha-linolenic acid (18:3 omega-3); DPA, docosapentaenoic acid (22:5 omega-3); EPA, eicosapentaenoic acid (20:5 omega-3); DHA, docosahexaenoic acid (22:6 omega-3).

^a^Statistically significant P-value < 0.05 in bold.

^b^Long-chain omega-3 PUFAs=DPA+EPA+DHA.

## Discussion

This cross-sectional study in Chinese women revealed reverse correlations between dietary or serum phospholipid long-chain omega-3 PUFAs (total- and long-chain- omega-3 PUFAs, EPA, DHA) and HOMA-IR in PCOS patients. Similarly, we found that, generally, dietary and serum phospholipid omega-3 PUFAs (total- and long-chain- omega-3 PUFAs, DPA, EPA, and DHA) were negatively associated with fat mass and body fat percentage, but positively correlated with muscle mass.

Prior evidence suggested that IR in ovarian tissue might appear to damage metabolic signaling but intact mitogenic and steroidogenic activity, which could further stimulate the production of ovarian androgens and aggravate hyperandrogenemia (28). Independent of BMI, hyperinsulinemia, which is closely related to personal lifestyle including inadequate nutrition accompanied by a lack of physical exercise, is a major component of PCOS pathogenesis (7). Diet is an effective, acceptable, and safe intervention for relieving IR (29). Regular consumption of fish and/or oral supplementation of omega-3 PUFAs are both key sources of omega-3 PUFAs in diet (30).

Prior studies showed a relationship between some omega-3 PUFAs and PCOS indices, though the effects of omega-3 PUFAs on insulin metabolism remain inconclusive. For instance, a double-blinded randomized placebo-controlled trial (RCT) among 30 pairs of PCOS patients and controls aged 18–40 years old suggested that a 12-week linseed oil omega-3 PUFAs supplementation (rich in DHA + EPA) significantly reduced insulin values and HOMA-IR, but increased insulin sensitivity check index (all *P*-values < 0.05) (17). Another RCT suggested that a combination of caloric restriction (1,200–1,500 kcal/day) and long-chain omega-3 PUFAs supplementation (DHA + EPA) significantly decreased the levels of HOMA-IR, insulin, and glucose-dependent insulinotropic polypeptide (GIP) secretion during the oral glucose tolerance test (OGTT) in obese subjects, but these effects were not seen with caloric restriction alone (31).

Yet, prior studies have yielded inconsistent effects of omega-3 PUFAs on body composition. An animal model indicated that treatment with flaxseed oil (rich in EPA and DHA) significantly reduced blood glucose and the amount of liver fat in rats fed with a high-fat diet (32). Of note, the present study did not observe the beneficial effect of ALA, but a prior cross-sectional study (33) involving 554 women aged 65–72 years found significant associations between dietary ALA and lower fat mass (β = –0.081, *P* = 0.034). This inconsistent result might be partly ascribed to the different portion size estimation or other biases related to subjective consumption assessment via different food record methods (3-day food record vs. 102 items food frequency questionnaire). Furthermore, given that there is no consistent evidence that omega-3 PUFA supplementation could be related to weight loss or reduced body fat mass in adults, (34) further researches are necessary to demonstrate the beneficial effect of dietary omega-3 PUFAs on body composition.

Although the exact etiology remains elusive, the beneficial effects of omega-3 PUFAs on insulin could be partly attributable to its role in insulin signaling and gene expression. Omega-3 PUFAs could protect glucose tolerance and avoid the accumulation of bioactive lipid mediators by up-regulating the mRNA expression of insulin-stimulated glucose transporter-4 (GLUT4), insulin receptor substrate-1 (IRS1), and glycogen synthase-1 (GYS1) (35). Additionally, by reducing endoplasmic reticulum stress, increasing β- oxidation of mitochondrial fatty acids and mitochondrial uncoupling, as well as limiting lipid deposits and reactive oxygen species generation, omega-3 PUFAs could further improve insulin sensitivity (16). DHA has been found to alleviate IR by regulating the pathway of the silent information regulator 1 (SIRT 1), a member of a protein family that could play a role in glucose homeostasis and IR reduction through lowering mitochondrial dysfunction (36). Additionally, IR and obesity are characteristics of chronic macrophage-mediated inflammation. Through signaling G-protein-coupled receptor 120 (GPR120), an omega-3 fatty acid receptor/sensor, omega-3 PUFAs could block both toll-like receptor (TLR) and TNF-inflammatory signaling pathways, then mediate M1–M2 macrophage polarization *via* down-regulating the expression of inflammatory genes (e.g., IL-6, TNF-a, MCP-1) but up-regulating the expression of anti-inflammatory genes (e.g., IL-10, MGL1, YM-1) in adipose tissue (37). Particularly, DHA has been found to decrease macrophage-derived inflammation and angiogenesis in adipose tissues, then reduce adipocyte size and body fat composition in middle-aged rats fed a high-fat diet (HFD) (36). Considering that most studies have been undertaken using animal and cellular models, whereas limited works have been conducted among humans, more high-quality studies are warranted to further explore the potential impact of omega-3 PUFAs on metabolic function in humans.

Concerning serum omega-3 PUFAs, we found that the HOMA-IR, fat mass and body fat percentages decreased as the circulating levels of total-, and long-chain omega-3 PUFAs (total-, DPA, EPA, and DHA) increased, but did not relate to ALA. However, the present study found a null association of total dietary omega-3 PUFA and its subtypes (EPA and DHA) with muscle mass, in accord with a study by Masoud Isanejad et al. (33). The inconsistent results between dietary and circulating omega-3 PUFAs may be attributable to the difference in concentration in omega-3 PUFA concentrations in the dietary and serum phospholipids (38). Because gastrointestinal digestion and the absorption process of foods could affect the bioavailability and clinical efficacy of dietary omega-3 PUFAs, leading to the underestimation of their beneficial effects (24). Furthermore, not only genetic factors could influence the molecular response to different sources of omega-3 PUFAs, but also epigenetic modifications could obscure the clinical benefits of omega-3 PUFAs (24).

The protective roles of omega-3 PUFAs on muscle cells are under-reported. A placebo-controlled, double-blind RCT among 28 boys with Duchenne muscular dystrophy (DMD) demonstrated that the blood levels of EPA and DHA in erythrocytes in patients who were supplemented with 2.9 g/d of long-chain omega-3 PUFAs for 6 months were both significantly higher than that of the placebo group (*P* < 0.05), and the supplementation could significantly slow the progression of muscle loss, lowered the fat mass and IR in DMD patients (39). An RCT conducted among 124 participants found that 16 weeks of omega-3 PUFA supplementation (containing 1,650 mg/day of DHA and 150 mg/day of EPA) significantly increased the quality of muscle in lower limbs in overweight/obese postmenopausal women (40).

The n-3 PUFAs could also counter pro-atrophic mediators in the muscle cells, such as maintaining the insulin receptor number, IRS-1 tyrosine phosphorylation, phosphatidylinositol (PI) 3′-kinase activity, and GLUT-4 content in rat muscle (41). A vitro study by Vigdis Aas et al. (42) found that glucose transport and oxidation (CO_2_) in muscle cells were increased by 2-folds and the glucose transporter-1 (GLUT1) expression was increased by 2.5-folds after preincubation of myotubes with 0.6 mM EPA for 24 h, indicating that exposure to EPA could stimulate the uptake and oxidation of glucose in skeletal muscle cells from young and healthy subjects. The supplementation of omega-3 PUFAs might improve muscle function, including increasing protein synthesis, improving metabolic function, and suppressing atrophy in muscle cells (43).

## Limitations

Even though this is the first study to comprehensively show that dietary and serum n-3 PUFAs might exert a positive effect on metabolism indexes and body composition among PCOS patients, several limitations need to be taken into consideration when interpreting our study. Firstly, the nature of cross-sectional epidemic research does not allow to draw of a causal relationship. Secondly, dietary intakes were self-reported, and therefore prone to some degree of misclassification. However, our trained investigators explained the dietary questions with common food pictures to help them estimate their dietary consumption. Thirdly, recall bias could not be ruled out as the patients were asked to recall their dietary patterns and other living habits over the past years. Nevertheless, to minimize the recall bias, face-to-face interviews were conducted with PCOS patients at the time when they were diagnosed. Fourthly, we cannot rule out selection bias because this is a hospital-based study. Finally, the conclusions were drawn from PCOS patients in Chengdu and Sichuan province in China, so our results may not be generalizable to other populations.

## Conclusion

In summary, this study found that higher levels of both dietary and serum omega-3 PUFAs, particularly long-chain omega PUFAs (DPA and DHA), might exert positive effects on metabolic parameters and body composition among PCOS patients. These findings may have important practical implications because they suggest that PCOS patients might gain health benefits from dietary supplementation of omega-3 PUFAs.

## Data availability statement

The datasets generated or analyzed during this study are available from the corresponding author on reasonable request.

## Ethics statement

The studies involving human participants were reviewed and approved by First Affiliated Hospital of Chengdu Medical College. The patients/participants provided their written informed consent to participate in this study.

## Author contributions

Formulating the research question(s): LLu. Designing the study: LLu, XL, and LLv. Carrying out the study: XL and LLv. Analyzing the data: YX, BW, and CH. Interpreting the findings: YX and CH. Writing the article: LLu, XL, and BW. All authors contributed to the article and approved the submitted version.

## Funding

This work was supported by Chengdu Medical Scientific Research project (No. 2021410), General Project of Natural Science Foundation of Xinjiang Uygur Autonomous Region (No. 2018D01C256), and Talent Research Startup Fund of Guangdong Second People's Hospital.

## Conflict of interest

The authors declare that the research was conducted in the absence of any commercial or financial relationships that could be construed as a potential conflict of interest.

## Publisher's note

All claims expressed in this article are solely those of the authors and do not necessarily represent those of their affiliated organizations, or those of the publisher, the editors and the reviewers. Any product that may be evaluated in this article, or claim that may be made by its manufacturer, is not guaranteed or endorsed by the publisher.
