# Corrigendum: Associations between omega-3 fatty acids and insulin resistance and body composition in women with polycystic ovary syndrome

**DOI:** 10.3389/fnut.2024.1383808

**Published:** 2024-03-01

**Authors:** Ling Lu, Xiaoqin Li, Lin Lv, Yao Xu, Baohua Wu, Chaolin Huang

**Affiliations:** ^1^Department of Gynecology, First Affiliated Hospital of Chengdu Medical College, Chengdu, China; ^2^Department of Oncology, Guangdong Second Provincial General Hospital, Guangzhou, China

**Keywords:** polycystic ovary syndrome, omega-3 polyunsaturated fatty acids, case-control studies, insulin resistance, body composition

In the published article, there was an error in affiliation 2. Instead of “Department of Oncology, Guangdong Second People's Hospital, Guangzhou, China,” it should be “Department of Oncology, Guangdong Second Provincial General Hospital, Guangzhou, China.”

The authors apologize for this error and state that this does not change the scientific conclusions of the article in any way. The original article has been updated.

