# Supplementary material for: Associations between omega-3 fatty acids and insulin resistance and body composition in women with polycystic ovary syndrome
Source: Front Nutr. 2022 Oct 5;9:1016943. doi: 10.3389/fnut.2022.1016943 (PMC9581053; doi:10.3389/fnut.2022.1016943)
Supplement: Supplementary file 1 [file Table_1.DOCX]

**sTable 1.** Associations between Omega-3 fatty acids and muscle mass measured by body composition analyzer among PCOS patients.

|  | **Muscle mass** | | |  | **Fat mass** | | |  | **Body fat percentage** | | |
| --- | --- | --- | --- | --- | --- | --- | --- | --- | --- | --- | --- |
|  | **Standardized coefficients β** | **SE** | ***P* value ^a^** |  | **Standardized coefficients β** | **SE** | ***P* value ^a^** |  | **Standardized coefficients β** | **SE** | ***P* value ^a^** |
| **Dietary omega-3 PUFA intakes** |  |  |  |  |  |  |  |  |  |  |  |
| Total omega-3 PUFAs (g/day) | 0.009 | 0.006 | 0.135 |  | -0.011 | 0.011 | 0.319 |  | -0.015 | 0.012 | 0.213 |
| Long-chain omega-3 PUFAs (mg/day) ^b^ | 0.015 | 0.008 | 0.062 |  | -0.022 | 0.011 | **0.047** |  | -0.026 | 0.012 | **0.032** |
| ALA (g/day) | 0.004 | 0.007 | 0.568 |  | -0.005 | 0.008 | 0.533 |  | -0.006 | 0.009 | 0.506 |
| DPA (mg/day) | 0.009 | 0.005 | 0.073 |  | -0.009 | 0.012 | 0.454 |  | -0.011 | 0.015 | 0.464 |
| EPA (mg/day) | 0.012 | 0.008 | 0.135 |  | -0.018 | 0.013 | 0.168 |  | -0.019 | 0.014 | 0.176 |
| DHA (mg/day) | 0.017 | 0.009 | 0.060 |  | -0.017 | 0.009 | 0.060 |  | -0.021 | 0.011 | 0.058 |
| **Serum phospholipid omega-3 PUFAs, %** |  |  |  |  |  |  |  |  |  |  |  |
| Total omega-3 PUFAs | 0.018 | 0.010 | 0.073 |  | -0.029 | 0.014 | **0.040** |  | -0.045 | 0.017 | **0.009** |
| Long-chain omega-3 PUFAs ^b^ | 0.023 | 0.011 | **0.038** |  | -0.059 | 0.015 | **<0.001** |  | -0.067 | 0.018 | **<0.001** |
| 18:3 omega-3 | 0.008 | 0.009 | 0.375 |  | -0.016 | 0.019 | 0.401 |  | -0.021 | 0.021 | 0.319 |
| 22:5 omega-3 (DPA) | 0.012 | 0.011 | 0.277 |  | -0.031 | 0.015 | **0.040** |  | -0.039 | 0.016 | **0.016** |
| 20:5 omega-3 (EPA) | 0.022 | 0.012 | 0.068 |  | -0.038 | 0.016 | **0.019** |  | -0.044 | 0.016 | **0.007** |
| 22:5 omega-3 (DHA) | 0.021 | 0.009 | **0.021** |  | -0.046 | 0.013 | **0.001** |  | -0.053 | 0.014 | **<0.001** |

**Abbreviations**: PCOS, polycystic ovary syndrome; SE, standard error; PUFA, polyunsaturated fatty acids; ALA, alpha-linolenic acid (18:3 omega-3); DPA, docosapentaenoic acid (22:5 omega-3); EPA, eicosapentaenoic acid (20:5 omega-3); DHA, docosahexaenoic acid (22:6 omega-3).

**^a^** Statistically significant *P*-value < 0.05.

**^b^** Long-chain omega-3 PUFAs=DPA+EPA+DHA.
